# Survival benefits of pelvic lymphadenectomy versus pelvic and para-aortic lymphadenectomy in patients with endometrial cancer: A meta-analysis

**DOI:** 10.1097/MD.0000000000009520

**Published:** 2018-01-05

**Authors:** Weina Guo, Jing Cai, Min Li, Hongbo Wang, Yi Shen

**Affiliations:** Department of Obstetrics and Gynecology, Union Hospital, Tongji Medical College, Huazhong University of Science and Technology, Wuhan, China.

**Keywords:** endometrial cancer, lymphadenectomy, meta-analysis, para-aortic, pelvic, survival

## Abstract

**Background:**

Despite that pelvic and para-aortic lymphadenectomy (PPaLND) is recommended as part of accurate surgical staging by International Federation of Gynecology and Obstetrics (FIGO) in endometrial cancer, the impact of para-aortic lymphadenectomy on survival remains controversial. The aim of this work is to evaluate the survival benefits or risks in endometrial cancer patients who underwent surgical staging with or without para-aortic lymphadenectomy using meta-analysis.

**Methods:**

Literature search was undertaken using PubMed, Embase, and Cochrane Library databases for relevant articles published between January 1, 1990, and January 1, 2017, without language restriction. The primary outcome was overall survival (OS); progression-free survival (PFS)/recurrence-free survival (RFS)/disease-free survival (DFS)/disease-related survival (DRS) was also analyzed. Subgroup analysis and sensitivity analysis were conducted to investigate the source of heterogeneity. Quality assessments were performed by Newcastle–Ottawa Quality Assessment Scale (NOS). Publication bias was evaluated by using Begg and Egger tests. The hazard ratio (HR) was pooled with random-effects or fixed-effects model as appropriate.

**Results:**

Eight studies with a total of 2793 patients were included. OS was significantly longer in PPaLND group than in pelvic lymphadenectomy (PLND) group for patients with endometrial cancer [HR 0.68; 95% confidence interval (CI) 0.55–0.84, *P* < .001, *I*^*2*^ = 12.2%]. Subgroup analysis by recurrence risk explored the same association in patients at intermediate- or high-risk (HR 0.52; 95% CI 0.39–0.69, *P* < .001, *I*^*2*^ = 41.4%), but not for low-risk patients (HR 0.48; 95% CI 0.21–1.08, *P* = .077, *I*^*2*^ = 0). PPaLND with systematic resection of all para-aortic nodes up to renal vein also improved PFS/RFS/DFS/DRS, compared with PLND (HR 0.52, 95% CI 0.37–0.72, *P* < .001, *I*^*2*^ = 0). No publication bias was observed among included studies.

**Conclusion:**

PPaLND is associated with favorable survival outcomes in endometrial cancer patients with intermediate- or high-risk of recurrence compared with PLND, particularly with regards to OS. PPaLND with systematic resection of all para-aortic nodes up to renal vein also improve PFS compared with PLND. Further large-scale randomized clinical trials are required to validate our findings.

## Abstract 2

Supplemental Digital Content is available in the text

## 1 Introduction

Endometrial carcinoma is the most common gynecological malignancy in developed countries.^[1]^ According to the estimate of the American Cancer Society, it was expected that there would be 61,380 new cases of uterine corpus cancers occurred and led to 10,920 deaths in 2017.^[2]^ The World Health Organization reported in 2012 that 319,605 cases of uterine corpus cancers have newly increased and led to 76,160 deaths worldwide.^[3]^ The management of endometrial cancer has significantly changed over the past 25 years.^[4,5]^ In primary surgery, surgical assessment of lymph nodes for staging remains one of the most varied practices worldwide,^[6]^ ranging from no nodal assessment, to sentinel lymph node (SLN) mapping, to complete pelvic and para-aortic lymphadenectomy up to the renal vessels.

Retroperitoneal lymph node metastasis is a critical prognostic factor for patients with endometrial cancer,^[7]^ approximately 10% of women with presumed early-stage endometrial cancer suffered lymph node metastasis.^[8]^ SLN mapping and biopsy is increasingly used in many gynecological centers, but the technique is still under investigation in order to improve the accuracy of SLN detection.^[9]^ Extensive lymphadenectomy may have therapeutic benefit due to the removal of occult metastasis that remains undiagnosed by the pathologist. However, the prognostic significance of routine dissection of the retroperitoneal para-aortic lymph node is still debated.^[10–12]^ Notably, retroperitoneal para-aortic lymph node dissection could increase the surgical morbidity.^[13]^ Compared with patients undergoing pelvic lymphadenectomy (PLND) alone, patients undergoing pelvic and para-aortic lymphadenectomy (PPaLND) suffered increased blood loss, transfusion rates, hospital stay, and anesthesia time.^[14]^ The prognostic information provided by para-aortic lymphadenectomy is useful; however, the tangible costs for gathering this information should also be considered. We aimed to perform an analysis of a total evidence of relevant studies evaluating the survival benefits or risks in endometrial cancer patients who underwent surgical staging with or without para-aortic lymphadenectomy.

## 2 Materials and methods

### 2.1 Search strategy

Literature search was undertaken using PubMed, Embase, and Cochrane Library databases for relevant articles published between January 1, 1990, and January 1, 2017, without language restriction. This study was prepared according to the recommendations of the Cochrane Collaboration and is reported following the Preferred Reporting Items for Systematic Reviews and Meta-analysis (PRISMA) Statement. The searching terms were (para-aortic) AND (pelvic) AND (“lymph node excision” [Mesh] OR “lymphadenectomy”[All Fields]) AND (“endometrial neoplasms” [Mesh] OR “endometrial cancer” [All Fields]) without limiting publication language. Reference lists of all available clinical studies were manually searched and reviewed.

### 2.2 Inclusion and exclusion criteria

We designed the meta-analysis to compare PLND and PPaLND surgical procedures for patients with EC. Inclusion criteria were as follows: studies exploring endometrial cancer; studies comparing 2 treatment modalities and reporting overall survival (OS)/progression-free survival (PFS)/recurrence-free survival (RFS)/disease-free survival (DFS)/disease-related survival (DRS); studies providing hazard ratio (HR) directly or key information to calculate HR indirectly, such as Kaplan-curves and original survival data. The following studies that met one of the criteria would be excluded: abstracts of meetings, duplicate publication, review articles, and case reports; studies explored new surgical techniques or evaluated operation outcomes without comparison of survival effects; and full text or valid data not accessed. To avoid overlapping patient data in publications on the same cohort, we included articles with the latest data.

### 2.3 Study selection and data extraction

Two reviewers (W.N.G. and J.C.) individually screened the electronic database according to the prespecified strategies. Disagreement was resolved through independently extracting data from the original article by the corresponding author, and consensus was reached by discussions. Several essential information was extracted, including first author, publication year, country of origin, number of patients analyzed, mean age, International Federation of Gynecology and Obstetrics stage (FIGO), median lymph nodes removed, follow-up time, outcome, recurrence risk categories and its criteria, extracting method of HR, and whether multiple analysis or not. The main features of these eligible studies are summarized in Table 1.^[15–17]^ For prognostic studies, when both multivariate and univariate analyses of the OS/PFS/RFS/DFS/DRS results were performed, HRs and their corresponding 95% confidence intervals (95% CIs) were extracted preferentially from the multivariate analyses. For the articles in which prognosis was plotted only as the Kaplan–Meier curves, the Engauge Digitizer 9.5 (Torrance, CA; http://markummitchell.github.io/engauge-digitizer) was then used to extract survival data, and the estimates of the HRs and 95% CIs were calculated by Tierney method.^[18]^

**Table 1:**
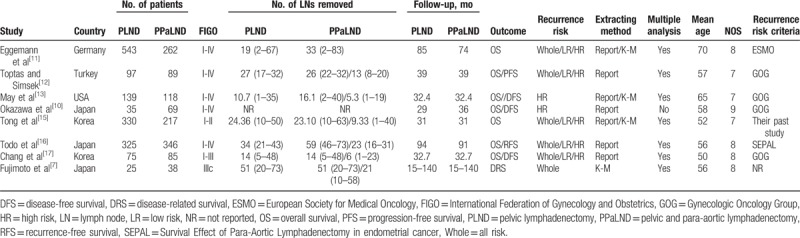
Basic characteristic of included studies.

### 2.4 Quality assessment

Quality assessments were performed according to the Newcastle–Ottawa Quality Assessment Scale (NOS),^[19]^ which contains 3 aspects: selection, comparability, and outcome. The highest quality studies are awarded up to 9 stars. Studies with more than 6 stars were considered as of high quality. Otherwise, studies were excluded from the final meta-analysis.

### 2.5 Statistical analysis

Statistical analysis was performed using STATA version 14.0 software (Stata Corporation, College Station, TX). The HR with 95% CI for data of survival analysis. Forest plots were generated for graphical presentations, and heterogeneity among different studies was appraised by Q statistics and *I*^*2*^ estimates. Fixed effects model was conducted to aggregate data if there were no statistical heterogeneity (*I*^*2*^ < 50%); otherwise, a random effects model would be considered. Sensitivity analysis was performed to identify the origin of any heterogeneity by sequentially eliminating individual studies. Publication bias was examined using Begg funnel plot and Egger linear regression test. Two-sided *P* < .05 was considered statistically significant.

### 2.6 Ethics approval

The ethical approval was not necessary in this study because of the meta-analysis study design.

## 3 Results

### 3.1 Characteristics of included studies

**Figure 1:**
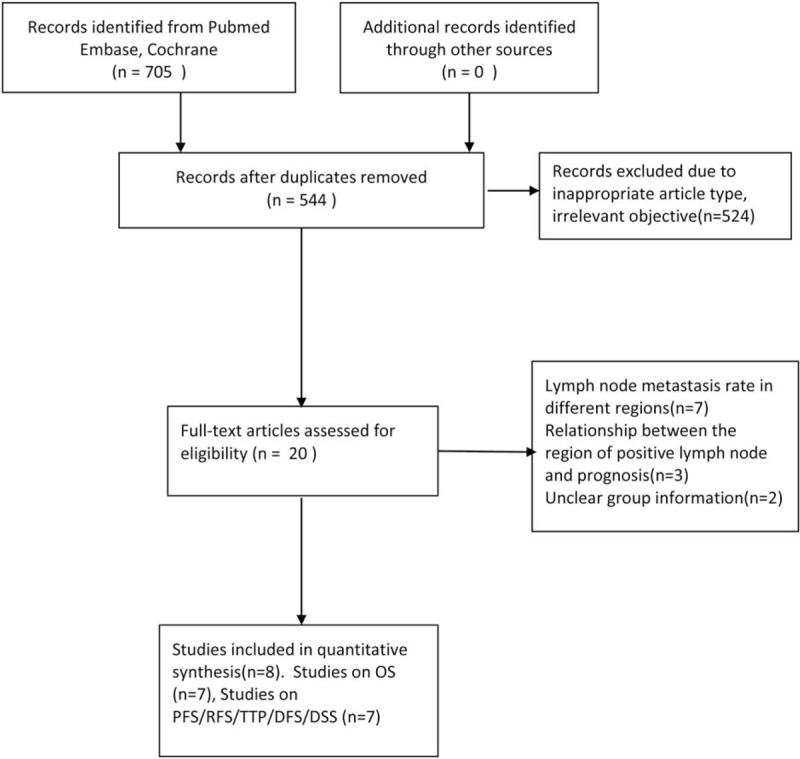
Articles searching flow chart.

A total of 544 records were searched after duplicates removed. Figure 1 outlines the selection process. Eight retrospective cohort studies were eligible for this meta-analysis that assessed prognosis between PPaLND and PLND in patients with endometrial cancer.^[7,10–13,15–17]^ A total of 2793 patients were included, 1456 patients received PLND alone, whereas 1337 patients received PPaLND. Characteristics of the 8 eligible studies are listed in Table 1. The included studies were published between 2007 and 2016. Four studies were studies were conducted in Japan,^[7,10,16]^ 3 in Korea,^[15,17]^ 1 in Turkey,^[12]^ 1 in Germany,^[11]^ 1 in USA.^[13]^ The average of follow-up duration was 46 months. We adopted NOS to assess the quality of included papers (http://www.ohri.ca/programs/clinical_epidemiology/oxford.asp). The quality score ranged from 7 to 9 with a median score of 8 for all cohort studies, which suggested the relatively high quality of the studies included in the meta-analysis (Table 1).

### 3.2 Results of meta-analysis

#### 3.2.1 Forest plot of HR for OS

**Figure 2:**
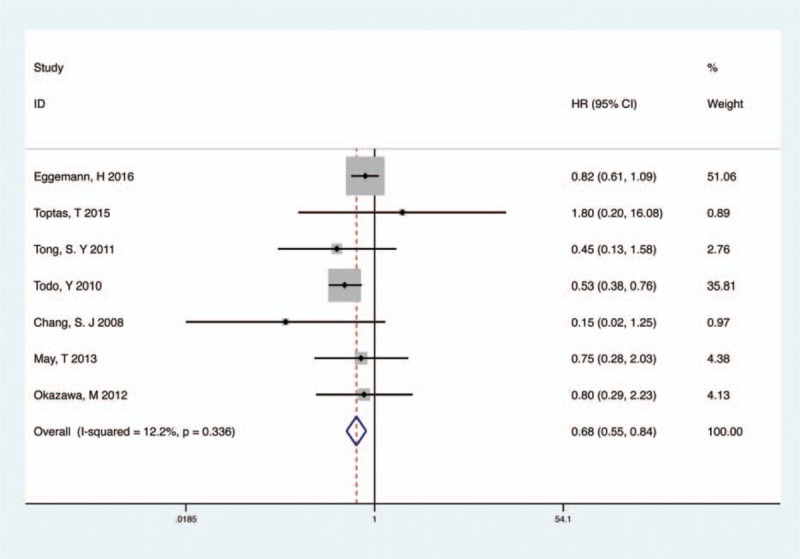
Meta-analysis of PPaLND and PLND on OS in endometrial cancer. OS = overall survival, PLND = pelvic lymphadenectomy, PPaLND = pelvic and para-aortic lymphadenectomy.

Seven studies involved 2730 endometrial patients and reported OS with PPaLND and compared with PLND^[10–13,15–17]^ (Fig. 2). No significant heterogeneity was detected across the studies (*P* = .336, *I*^*2*^ = 12.2%), so the fixed-effect model was used in this analysis. The pooled HR (HR = 0.68, 95% CI 0.55–0.84, *P* < .001) suggested that PPaLND improved OS, compared with PLND, in all stages of endometrial cancer. The similar pooled HR 0.67 with 95% CI 0.56 to 0.86 was detected in the random-effect model.

**Figure 3:**
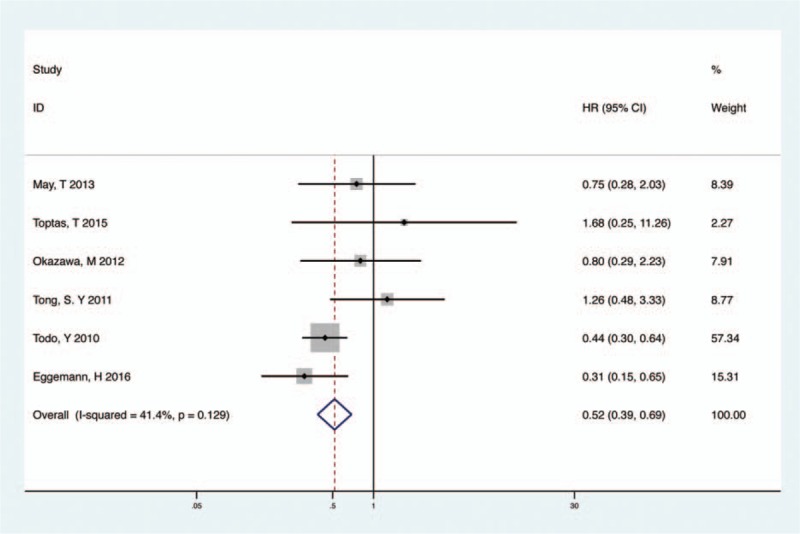
Meta-analysis of PPaLND and PLND on OS in intermediate- or high-risk patients. OS = overall survival, PLND = pelvic lymphadenectomy, PPaLND = pelvic and para-aortic lymphadenectomy.

On the basis of the recurrent risk evaluated intra- or postoperatively, we analyzed 2 recurrence rate groups (low-risk OS, intermediate- or high-risk OS) between PPaLND and PLND. Recurrent risk is related to the depth of myometrial invasion, tumor grade, histological subtype, and lymph-vascular space invasion in clinically proven early-stage endometrial cancer.^[16]^ These prognostic factors are aggregated to define patients with high recurrence risk and therefore are included in the risk stratification systems of European Society for Medical Oncology (ESMO), Gynecologic Oncology Group (GOG), Survival Effect of Para-Aortic Lymphadenectomy in endometrial cancer (SEPAL), and Post-Operative Radiation Therapy in Endometrial Carcinoma (PORTEC 1) in varies combination.^[6]^ Therefore, we defined patients with endometrial cancer of FIGO stage IA, grade 1 to 2, endometrioid histology, and negative lymphovascular space invasion (LVSI) as the low-risk group and the remaining patients as the intermediate- or high-risk group. Six studies involved 1729 intermediate- or high-risk endometrial cancer patients and compared PPaLND versus PLND.^[10–13,15,16]^ There was no heterogeneity among the included studies (*P* = .129, *I*^*2*^ = 41.4%) and a fixed-effect model was used. As listed in Fig. 3, PPaLND improved OS when compared with PLND in patients with intermediate- or high-risk of recurrence (HR 0.52; 95% CI 0.39–0.69, *P* < .001, *I*^*2*^ = 41.4% Fig. 3), but not for low-risk patients (HR 0.48; 95% CI 0.21–1.08, *P* = .077, *I*^*2*^ = 0 Fig. S1).

#### 3.2.2 HR for PFS/RFS/DFS/DRS and subgroup analysis

**Figure 4:**
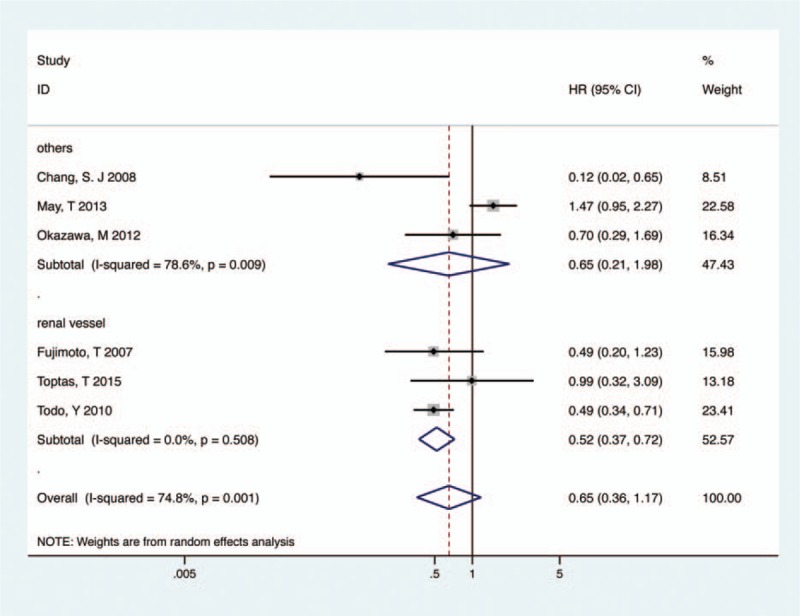
Meta-analysis of PPaLND and PLND on PFS/RFS/DFS/DRS in endometrial cancer. DFS = disease-free survival, DRS = disease-related survival, PFS = progression-free survival, PLND = pelvic lymphadenectomy, PPaLND = pelvic and para-aortic lymphadenectomy, RFS = recurrence-free survival.

Highly significant heterogeneity (*I*^*2*^ = 74.8%, *P* = .001) was detected when 6 studies were adopted to pool HRs for PFS/RFS/DFS/DRS.^[7,10,12,13,16,17]^ To make a conservative estimate, a random-effect model was used to account for the highly significant inter-study heterogeneity. Despite the lack of significant difference, PPaLND had a tendency to improve PFS in all stage endometrial cancer (HR 0.65, 95% CI 0.36–1.17, *P* = .152, *I*^*2*^ = 74.8%, Fig. 4).

Given that the substantial heterogeneity exhibited in the trials, subgroup analysis about the extent of para-aortic lymphadenectomy was conducted to explore the heterogeneity. When systematic resection of all para-aortic node up to renal vein, PPaLND improved PFS, compared with PLND (HR 0.52, 95% CI 0.37–0.72, *P* < .001, *I*^*2*^ = 0, Fig. 4), while there was no difference in PFS between unsystematic PPaLND and PLND (HR 0.65, 95% CI 0.21–1.98, *P* = .446, *I*^*2*^ = 78.6%, Fig. 4).

Moreover, there was no significant difference in PFS between PPaLND and PLND either in intermediate- or high-risk group (HR 0.71, 95% CI 0.36–1.40, *P* = .322, *I*^*2*^ = 78.8%, Fig. S2) or in low-risk group (HR 0.62, 95% CI 0.27–1.43, *P* = .26, *I*^*2*^ = 0, Fig. S3).

### 3.3 Sensitivity analysis

**Figure 5:**
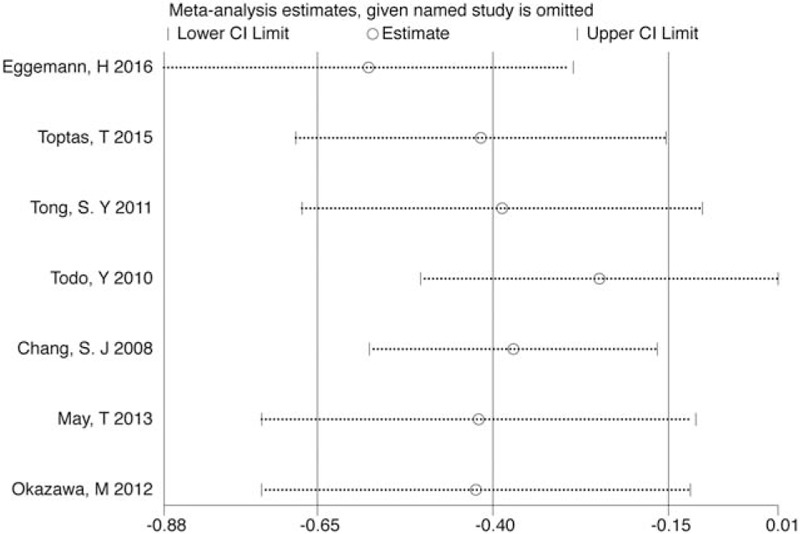
Sensitivity analysis for testing HR for OS. HR = hazard ratio, OS = overall survival.

Sensitivity analysis was performed to identify the origin of any heterogeneity by sequentially eliminating individual studies. The pooled HRs of remaining studies slightly differed with each other, but they did not change the final trend across sensitivity analysis. The results indicated that there was not a single study that significantly contributed to heterogeneity both for OS (Fig. 5) and PFS/RFS/DFS/DRS (Fig. S4).

### 3.4 Publication bias

**Figure 6:**
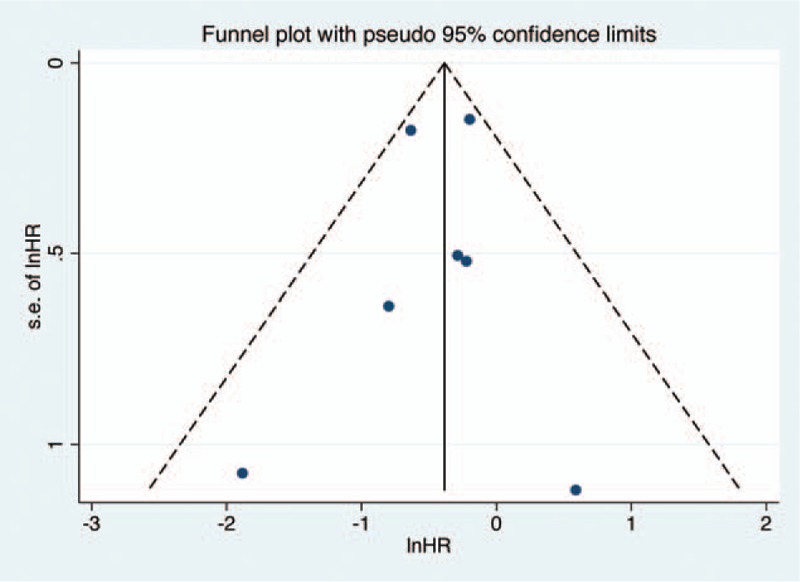
Funnel plots to evaluate publication bias of included studies for OS. OS = overall survival.

Egger test and Begg test were used to detect publication bias in the meta-analysis. A funnel plot was made for visual screening of any publication bias in the meta-analysis of OS (Fig. 6). It revealed that all studies were distributed evenly across the graph, suggesting no obvious publication bias in this meta-analysis. The *P* value of Egger and Begg tests were .168 and .881, respectively, showing no evidence of significant publication bias.

## 4 Discussion

Lymph node spread represents the most common site of extrauterine disease in endometrial cancer.^[20]^ Identification of patients with nodal metastases using surgical staging is important for providing guidance on adjuvant treatment and prognosis. Although surgical staging is the standard operation method of endometrial cancer, the extent and benefit of lymphadenectomy remain controversial.^[21]^ Previous studies have emphasized that the detection of occult lymph node metastasis was important for predicting prognosis, showing that 4.1% to 5.6% of the patients with low-risk endometrial cancer may have lymph node metastasis on the final histologic reports.^[22,23]^ In patients with intermediate- or high-risk endometrial cancer, lymph node metastasis was observed in 21.9% of the patients, of which, para-aortic lymph node involvement rate up to 15%.^[21]^ Moreover, a growing number of studies found that the skip metastasis, namely isolated para-aortic lymph node metastasis in the setting of negative pelvic nodes, has been reported in less than 5% of patients but mostly associated with risk factors for lymph node involvement.^[21,24]^ The extent of para-aortic lymphadenectomy varies greatly among study groups. A survey of gynecologic oncologists conducted by Soliman et al^[25]^ found that 50% of surgeons used the inferior mesenteric artery as the upper border, and only 11% extended the dissection to the renal vessels, whereas several anatomic data and lymphatic mapping studies clearly supported that para-aortic lymph node above the inferior mesenteric artery directly drain the uterine body, which is associated with lymph nodes metastasis. However, among all studies referring to the survival benefit of PPaLND compared with PLND, there existed some contradictory points requiring adequate attention, so, a comprehensive study is therefore urgent.

To the best of our knowledge, this is the first meta-analysis focused on the comparison of PPaLND versus PLND in endometrial cancer. Previously, a meta-analysis compared OS between systematic lymphadenectomy and unsystematic lymphadenectomy and concluded that systematic lymphadenectomy significantly improved OS of the endometrial cancer patients with intermediate- and high-risk of recurrence, but it did not focus on the para-aortic lymph node.^[26]^ Two large trials of PLND, that is, the ASTEC (A Study in the Treatment of Endometrial Cancer) trial^[27]^ and the study by Benedetti Panici et al,^[28]^ did not show any significant survival benefit of PLND in women with early endometrial cancer. However, less than 10 lymph nodes were removed in 35% of the patients in the lymphadenectomy group and neither study included para-aortic lymphadenectomy, which may contribute to the discrepancy between their and our present results. The survival benefit of para-aortic lymphadenectomy remains unclear. In this study, we calculated the pooled HRs for both OS and PFS, respectively, to obtain convincing results. On the whole, we identified that PPaLND was efficient for improving OS in all stage endometrial cancer patients compared with PLND. Exploratory analysis also suggested that the efficacy of PPaLND is limited in low-risk endometrial cancer, whereas it is efficient to increase OS in patients with intermediate- or high-risk endometrial cancer. For PFS, there was no significant difference between PPaLND and PLND. However, on the basis of the outcome of subgroup analysis, systematic PPaLND, namely systematic resection of all para-aortic node up to renal vein, improved PFS in all stage of endometrial cancer, compared with PLND. The result indicated that the extent of para-aortic lymphadenectomy may impose a great impact on PFS. It is worth mentioning that 1 study found that para-aortic lymphadenectomy yielding less than 10 nodes was associated with decreased PFS.^[13]^ This was a striking resemblance to our findings—3 studies undergoing systematic PPaLND yielded more than 10 para-aortic lymph nodes. The number of lymph node harvested would be an index of the degree of thoroughness in removal.^[29]^ But there were not enough studies to quantify the relationship between the number of para-aortic lymph node removed and the prognosis. Although using a cutoff at the median number of nodes is relatively arbitrary, the results illustrated that, besides the anatomy range of the operation, the number of para-aortic lymph node removed may be another important factor to measure the thoroughness of surgery.

In general, PPaLND could increase the risk of operative complications.^[14]^ However, a study found that intraoperative and postoperative complication rates were equivalent between PPaLND and PLND.^[13]^ SLN mapping is considered as an alternative. Studies revealed that SLN mapping provided similar detection rates of lymph node metastases in advanced stage of endometrial cancer^[30]^ and did not decrease DFS in patients with limited myometrial invasion.^[31]^ Therefore, future studies comparing SLN mapping versus systematic lymphadenectomy regarding the OS and DFS in patients with endometrial cancer should be taken into account.

The results of our study have some guiding significance for the grouping of patients with endometrial cancer and the selecting of corresponding surgical scope. However, there are some hidden biases in the retrospective cohort analysis. Perhaps patients who undergo PPaLND are more likely to be operated in tertiary medical centers by more accomplished gynecologic oncologists and received standard treatment. Moreover, the patients’ health and size may affect the decision of surgical approach. These biases may affect the analysis. Our findings in this review should be further verified in larger clinical trials with more extensive comparable data.

## 5 Conclusion

The present systematic review of retrospective cohort studies reveals that PPaLND is associated with favorable survival outcomes in endometrial cancer patients with intermediate- or high-risk of recurrence compared with PLND, particularly with regards to OS. PPaLND with systematic resection of all para-aortic node up to renal vein may also improve PFS compared with PLND. In summary, this study provides some guidance for choosing the appropriate surgical scope in the different groups of endometrial cancer patients. Due to the limitations inherent with retrospective studies, further large-scale randomized clinical trials are required to validate our findings.

## Supplementary Material

Supplemental Digital Content
