# Supplementary material for: Survival benefits of pelvic lymphadenectomy versus pelvic and para-aortic lymphadenectomy in patients with endometrial cancer: A meta-analysis
Source: Medicine (Baltimore). 2018 Jan 5;97(1):e9520. doi: 10.1097/MD.0000000000009520 (PMC5943115; doi:10.1097/MD.0000000000009520)
Supplement: Supplemental Digital Content [file medi-97-e9520-s001.doc]

**Figure S1.** Meta-analysis of PPaLND and PLND on OS in low-risk patients. PPaLND = pelvic and para-aortic lymphadenectomy, PLND = pelvic lymphadenectomy, OS = overall survival.

**
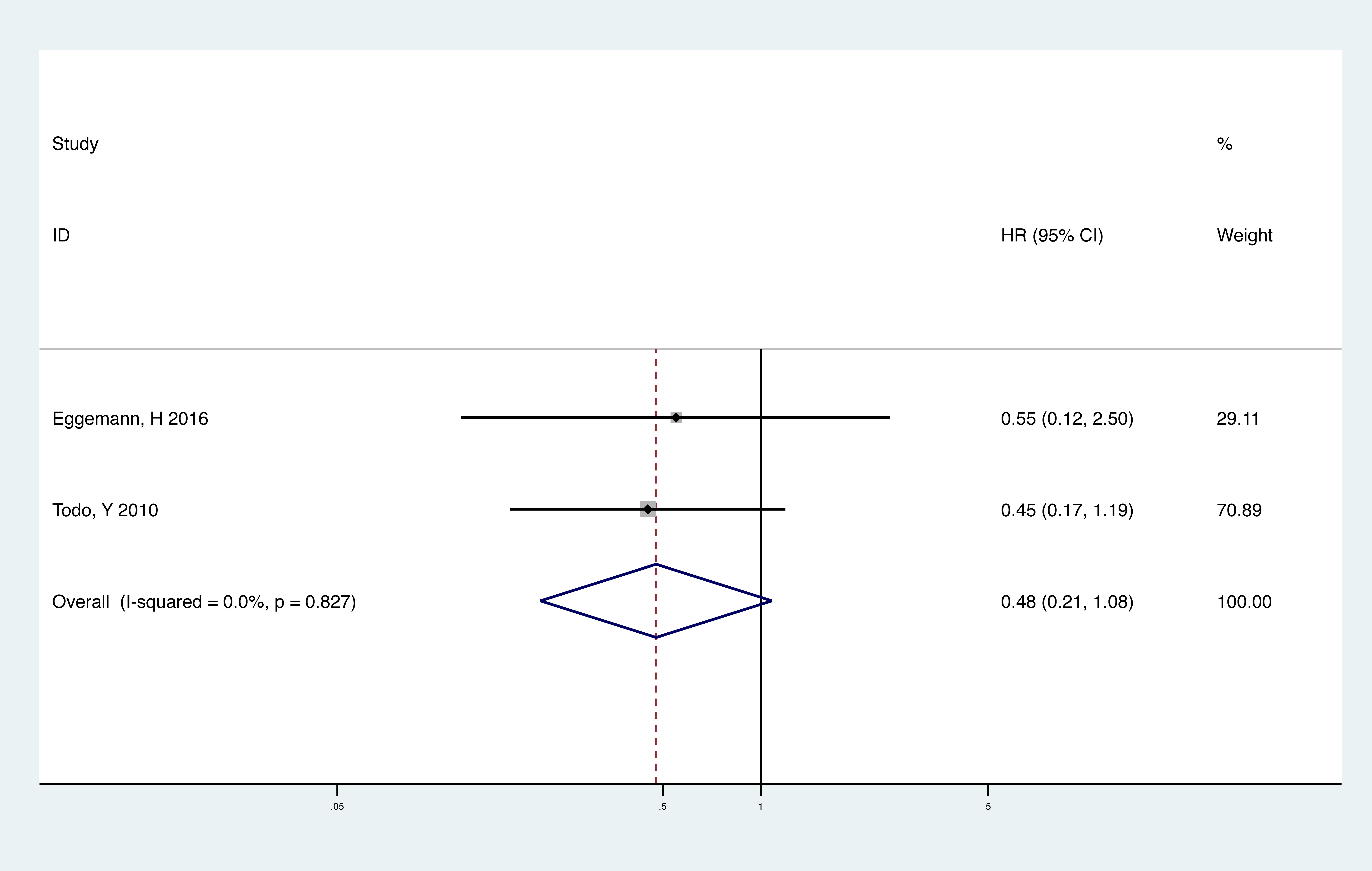
**

**Figure S2.** Meta-analysis of PPaLND and PLND on PFS/RFS/DFS/DRS in intermediate- or high-risk patients. PPaLND = pelvic and para-aortic lymphadenectomy, PLND = pelvic lymphadenectomy, PFS = progression-free survival, RFS = recurrence-free survival, DFS = disease-free survival, DRS = disease-related survival.

**
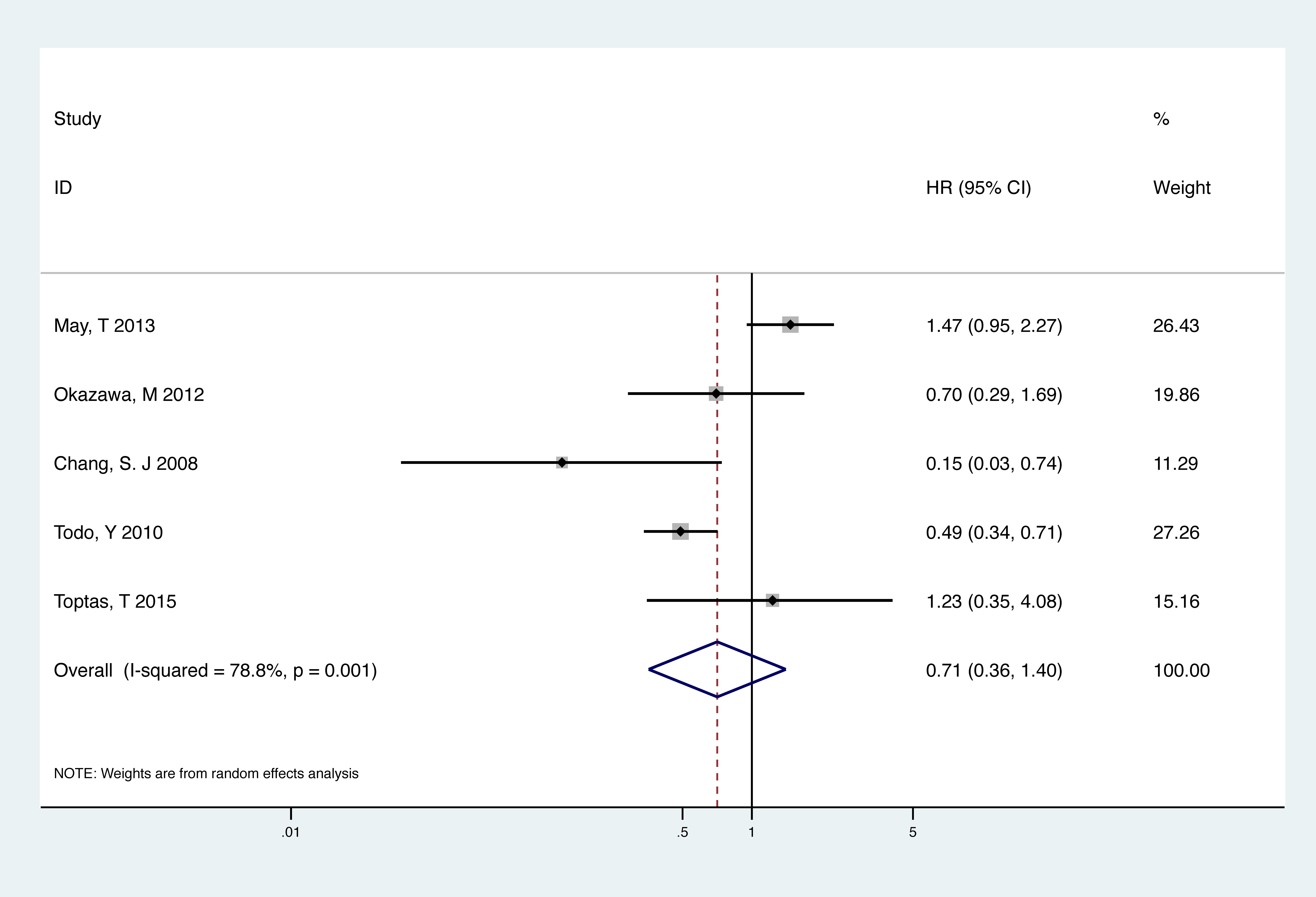
**

**Figure S3.** Meta-analysis of PPaLND and PLND on PFS/RFS/DFS/DRS in low-risk patients. PPaLND = pelvic and para-aortic lymphadenectomy, PLND = pelvic lymphadenectomy, PFS = progression-free survival, RFS = recurrence-free survival, DFS = disease-free survival, DRS = disease-related survival.

**
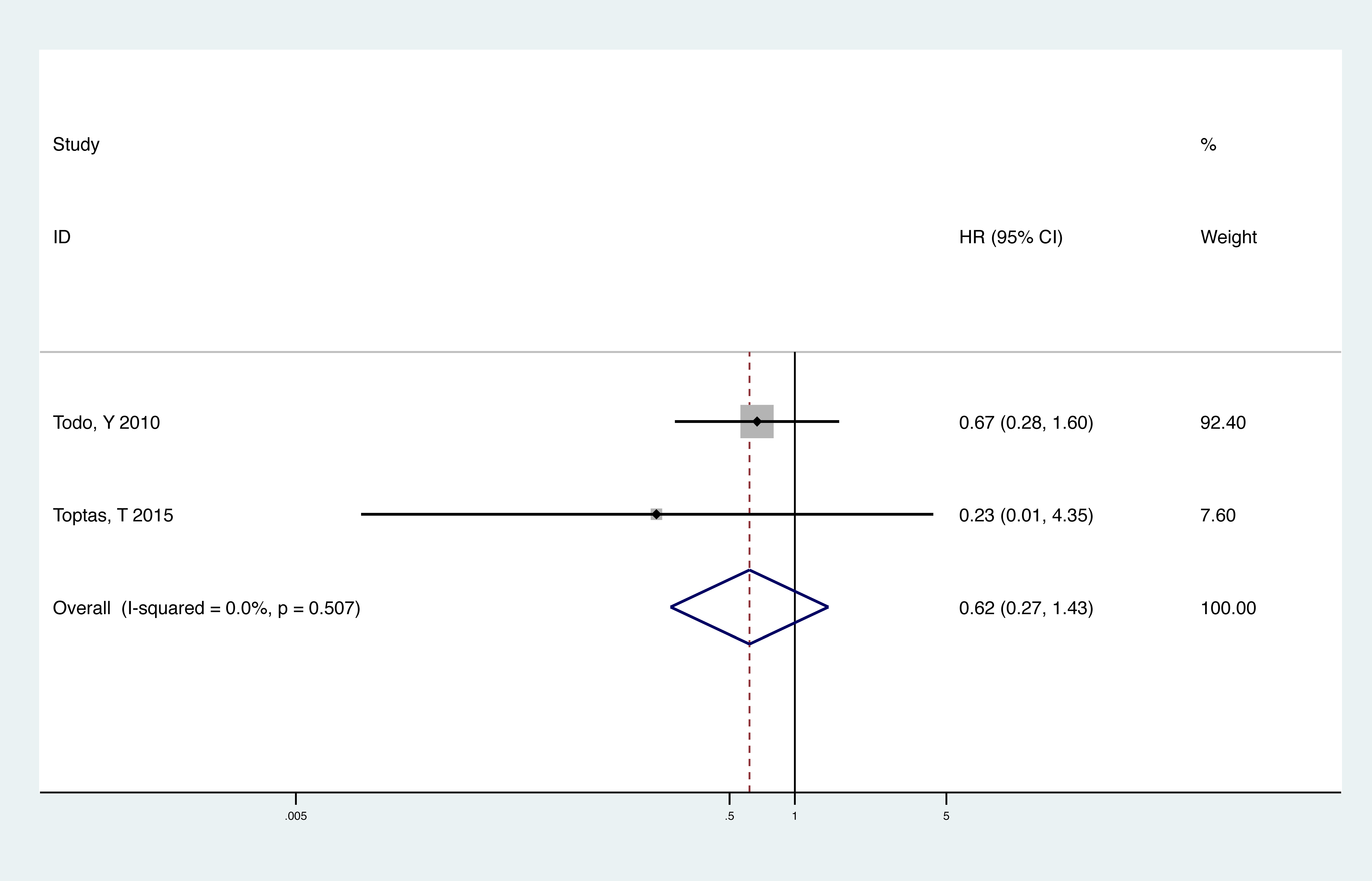
**

**Figure S4.** Sensitivity analysis for testing HR for PFS/RFS/DFS/DRS. HR = hazard ratio, PFS = progression-free survival, RFS = recurrence-free survival, DFS = disease-free survival, DRS = disease-related survival
